# Association between self-rated depressive symptoms and mucosal expression of NF-κ B in patients with upper gastrointestinal symptoms

**DOI:** 10.1186/s13030-023-00264-7

**Published:** 2023-02-25

**Authors:** Julia Staab, Lara Vonhören, Harald Schwörer, Thomas Meyer

**Affiliations:** 1 Department of Psychosomatic Medicine and Psychotherapy, University Medical Center Göttingen, Georg August University Göttingen, Waldweg 33, 37073 Göttingen, Germany; 2 Department of Paediatric Cardiology and Intensive Care Medicine, University Medical Center Göttingen, Georg August University, Göttingen, Germany; 3 Department of Gastroenterology, Gastrointestinal Oncology and Endocrinology, University Medical Center Göttingen, Georg August University, Göttingen, Germany

**Keywords:** NF-κ B, Depression, Gastritis, Inflammation, Upper gastrointestinal tract

## Abstract

**Background:**

Previous clinical studies have reported elevated levels of depressive symptoms in selected samples of patients with gastritis. The objective of this study was to examine the associations of specific biomarkers of inflammation expressed in mucosal tissue from the stomach with mood and anxiety symptoms in adult patients with upper gastrointestinal symptoms.

**Methods:**

In this monocentric, observational study, a total of 32 study participants were included who were referred for a routine diagnostic upper endoscopic assessment based on the suspected clinical diagnosis of gastritis. All participants completed the Hospital Anxiety and Depression Scale (HADS) before undergoing gastroscopy. Immunohistochemical stainings from biopsy sections were performed to evaluate the expression level of nuclear factor kappa B (NF-κ B), myeloperoxidase (MPO) and inducible nitric oxide synthase (iNOS).

**Results:**

Our findings confirmed that nearly half of the study cohort (*n* = 13; 41%) displayed positive HADS depression scores above the clinically relevant cut-off level of ≥ 8. Regression models demonstrated that depressive symptoms were significantly and positively associated with the expression level of NF-κ B in biopsies from the upper gastrointestinal tract.

**Conclusions:**

In summary, our study showed a significant association between NF-κ B expression and clinically relevant depressive symptoms in patients with gastritis, as assessed by a self-rated psychometric questionnaire. Further investigations are needed to confirm this relationship and to identify the pathophysiological mechanisms involved.

## Introduction

Numerous clinical studies have demonstrated elevated levels of depressive symptoms among selected samples of patients with gastritis [1–5]. In patients with *Helicobacter pylori-*induced gastritis, levels of interleukin (IL)-8 and tumour necrosis factor-α are increased in the gastric mucosa and the bacterial infection indirectly activates nuclear factor kappa B (NF-κ B) [6–8]. The transcription factor NF-κ B regulates multiple aspects of innate and adaptive immune functions and serves as a pivotal mediator of inflammatory responses [9]. Activation of NF-κ B and up-regulation of interleukin-8 (IL-8) in gastric epithelial cells have been suggested to be critical pathophysiological mechanisms responsible for *H. pylori*-induced chronic inflammation [10, 11].

Inflammatory conditions in the gastric mucosa are characterized by activation of NF-κ B, resulting in the expression of NF-κ B-regulated, inflammatory-inducible genes, such as inducible nitric oxide synthase (iNOS) and cyclo-oxygenase-2 (COX-2) [10]. Elevated expression of iNOS and increased nitric oxide synthesis are a hallmark of the inflammatory reaction in *H. pylori*-induced gastritis [12]. Studies have shown that a constitutive overproduction of iNOS and, hence, of nitric oxide may lead to DNA and protein damage, which could increase the risk of development of cancer [13]. Another histopathological feature of chronic gastritis caused by *H. pylori* infection is the infiltration of neutrophils into the gastric mucosa. Mucosal biopsy specimens from patients with *H. pylori* infection showed increased amounts of reactive oxygen species (ROS) and expression of myeloperoxidase (MPO), which catalyses the production of hypochlorous acid (HOCl) from hydrogen peroxide (H_2_O_2_) and chloride anion [14, 15]. The heme enzyme MPO is localized in lysosomes of infiltrating neutrophils, macrophages, and monocytes, and makes MPO activity into a potential biomarker of gastric inflammation.

Although previous studies have indicated an association between upper gastrointestinal symptoms and mood disorders, no previous investigation has examined the link between the expression of histopathological biomarkers of gastritis and depressive symptoms in humans. The aim of this pilot study was to determine the immunohistochemical expression patterns of NF-κ B, iNOS and MPO in biopsies from the upper gastrointestinal tract and their relationships to depressive symptoms.

## Materials and methods

### Study design

This is an observational, monocentric cohort study that used data from 32 participants who had been admitted to the Department of Gastroenterology at the university hospital of Göttingen, Germany, between July 2014 and September 2015. This clinical study included patients who had undergone a routine diagnostic upper endoscopic assessment based on the suspected clinical diagnosis of gastritis. All study participants were over 18 years and exhibited an existent medical indication for an endoscopic examination. Patients were excluded when they had inadequate knowledge of the German language or were unable to give consent due to the presence of predefined diagnosed psychiatric comorbidities, such as drug abuse or psychotic diseases. The study protocol was approved by the local ethics committee of the University Hospital of Göttingen (UMG-12/2/14). All study participants gave their written informed consent.

### Assessment of anxiety and depression

Anxiety and depression were measured using the Hospital Anxiety and Depression Scale (HADS). This is a self-completed questionnaire that has been validated and used in a variety of clinical settings [16, 17]. It consists of 14 items and is divided into an anxiety and a depression subscale, with a sum score ranging from 0 to 21 for each and a cut-off value of > 7 on either of the two subscales. Scores of 0–7 are considered normal, 8–10 are indicative of mild anxiety and depression symptoms, whereas higher scores above 10 are indicative of moderate or severe symptoms.

### Immunohistochemical staining

Histological data were obtained from biopsies taken during endoscopy. In nearly all study patients (*n* = 31, 96.8%), tissue biopsies were taken from the gastric antrum, and in 15 patients (46.9%) additional biopsies were obtained from the gastric corpus. Rarely were biopsy specimens collected from the cardia and fundus (*n* = 3, 9.4%) Each specimen was routinely fixed in 4% neutral buffered formalin and embedded in paraffin. Serial sections of 3 μm were cut from paraffin embedded tissue blocks and deparaffinized through a graded series of alcohol. Subsequently, the rehydrated sections were heat-treated for 15 min in citrate buffer followed by an incubation with peroxidase blocking solution for 15 min at 4 °C. For immunohistochemical detection of NF-κ B p65, a mouse monoclonal antibody (F-6, Santa Cruz), diluted 1:200 in 4% bovine serum albumin/phosphate-buffered saline (BSA/PBS) was used. iNOS was detected with a rabbit polyclonal antibody (H-174), diluted 1:200 in 4% BSA/PBS, which was obtained from Santa Cruz. For the detection of MPO, a rabbit polyclonal antibody (PA5-16672), diluted 1:200 in 4% BSA/PBS obtained from Thermo Scientific, was used. Detection of bound immunoglobulins was achieved with the ABC method employing biotinylated secondary antibodies and avidin-horseradish peroxidase complexes. Diaminobenzidine was used as a substrate for visualization of the enzyme reaction yielding a brown reaction product. Finally, the sections were counterstained with Mayer's haematoxylin, dehydrated and mounted. Imaging was performed with a light microscope (Zeiss). The staining intensity for each target antigen was evaluated using a four-point semiquantitative scoring system ranging from 1 (indicating virtually no immunostaining) to 4 (with the most prominent expression and infiltration). Histopathological assessment was performed by two investigators blinded to the clinical data from the cohort. The presence of *H. pylori* in the mucosal tissue specimens was tested by means of Giemsa staining and the urease-based *Campylobacter*-like organism test (CLO test). Given the greater diagnostic accuracy of the Giemsa test compared with the CLO test, only positive results of the Giemsa test were considered specific evidence of patients infected with *H. pylori*.

### Statistical analysis

Statistical analysis was undertaken using the IBM SPSS statistical software, version 22. The study cohort was characterized based on the assessment of anxiety and depression symptoms by means of the self-completed HADS questionnaire. Demographic and clinical data were expressed in terms of means ± standard deviations for continuous variables or frequencies and percentages for categorial variables. Differences between subgroups were assessed using chi-square tests for categorical measures and *t* tests for continuous measures. To assess the association of NF-κ B immunopositivity with psychometric data, Mann-Whitney U test and univariate ANOVA (analysis of variance) with Tukey’s post hoc analysis were performed. Logistic regression analyses were performed to determine whether associations existed between depression and the expression of NF-κ B. Multivariate analyses were performed with HADS-D ≥ 8 as the dependent variable and age, sex, NF-κ B and mean corpuscular volume (MCV) as confounding variables. Cox and Snell R^2^ was used as an indicator for the amount of explained variance in the regression models. In the logistic regression model, Exp(B) coefficients were calculated together with 95% confidence intervals (95% CI). Results from Wald tests are also reported. *P* values < 0.05 were regarded as statistically significant.

## Results

### Characterization of the study cohort

The sample comprised 32 patients who were treated at the Department of Gastroenterology and Endocrinology from July 2014 to September 2015 and had filled out the HADS questionnaire completely. Twenty-one out of the study sample were males (65.6%); the mean age of the study population was 54.3 $\pm$ 14.4 years. Thirteen study participants showed an elevated HADS depression scale (40.6%), whereas only four patients were categorized as having elevated HADS anxiety scores (12.5%). The main gastrointestinal symptoms in the study population were abdominal distension and/or inappetence (*n* = 12, 37.5%) followed by upper abdominal pain (*n* = 11, 34.4%), nausea (*n* = 6, 18.8%), dysphagia (*n* = 5, 15.6%), emesis (*n* = 3, 9.4%), dyspepsia (*n* = 2, 6.3%), and unspecific symptoms (*n* = 7, 21.9%). *Helicobacter pylori* infection was identified histochemically in 7 patients (4 males, 3 females). Positivity for *H. pylori* was neither associated with elevated HADS depression (*p* = 0.892) nor anxiety scores (*p* = 0.258) above the cut-off. In the group comparison, International Normalized Ratio (INR) was the only parameter that differed between depressed and non-depressed patients (*p* = 0.040). Demographic and clinical data of the study sample as stratified by HADS depression and anxiety scores are presented in Table 1.

**Table 1:** Characteristics of the study population undergoing gastroscopy stratified by abnormal HADS depression and anxiety scores (cut-off: HADS-A ≥ 11 = positive, HADS-D ≥ 8 = positive). Shown are frequencies (%) or means and standard deviations of the indicated variables as well as the corresponding *p* values for the comparisons between the two groups with normal and elevated HADS scores

	Total study population (*n* = 32)	Subjects with a HADS-D score above the cut-off (*n* = 13)	Subjects with a HADS-D score below the cut-off (*n* = 19)	*P*-value	Subjects with a HADS-A score above the cut-off (*n* = 4)	Subjects with a HADS-A score below the cut-off (*n* = 28)	*P*-value
Sex [male, %]	65.6	42.9	57.1	0.722	14.3	85.7	0.673
Age [years]	54.3 ± 14.4	56.3 ± 12.4	52.4 ± 15.4	0.448	62.3 ± 9.0	52.8 ± 14.4	0.216
Quick [%]	95.2 ± 20.0	85.1 ± 16.1	102.2 ± 19.9	**0.026**	84.5 ± 24.9	97.1 ± 19.1	0.253
INR	1.1 ± 0.1	1.1 ± 0.1	1.0 ± 0.1	**0.040**	1.2 ± 0.2	1.0 ± 0.1	0.083
Hb [g/dl]	13.0 ± 2.0	13.3 ± 2.3	12.8 ± 1.7	0.511	12.7 ± 3.3	13.1 ± 1.8	0.691
Hct [%]	39.1 ± 5.6	39.9 ± 6.7	38.5 ± 4.7	0.510	37.8 ± 9.2	39.3 ± 5.1	0.628
Erythrocytes [10^6^/µl]	4.4 ± 0.7	4.6 ± 0.7	4.3 ± 0.7	0.183	4.3 ± 0.9	4.4 ± 0.7	0.824
MCV [fl]	89.0 ± 7.3	86.6 ± 6.6	90.6 ± 7.5	0.130	87.0 ± 6.9	89.3 ± 7.5	0.568
MCH [pg]	29.7 ± 2.7	28.9 ± 2.2	30.3 ± 3.0	0.186	29.1 ± 2.6	29.7 ± 2.8	0.646
MCHC [g/dl]	33.3 ± 0.8	33.4 ± 0.6	33.2 ± 0.9	0.717	33.4 ± 0.6	33.3 ± 0.8	0.805
Platelets [10^3^/µl]	242.5 ± 115.5	239.7 ± 146.1	244.5 ± 93.5	0.911	168.8 ± 76.7	253.1 ± 117.2	0.176
Leukocytes [10^3^/µl]	7.1 ± 2.4	6.8 ± 2.2	7.2 ± 2.6	0.646	6.6 ± 2.8	7.2 ± 2.4	0.645
Na^+^ [mmol/l]	138.5 ± 3.6	137.7 ± 5.0	139.1 ± 1.9	0.349	136.0 ± 8.6	138.9 ± 2.2	0.551
K^+^ [mmol/l]	4.0 ± 0.5	4.1 ± 0.6	4.0 ± 0.4	0.493	3.9 ± 0.5	4.1 ± 0.5	0.557
Cr [mg/dl]	0.9 ± 0.4	1.0 ± 0.5	0.9 ± 0.4	0.633	1.0 ± 0.5	0.9 ± 0.4	0.809
eGFR	55.7 ± 8.8	54.3 ± 11.1	56.9 ± 6.3	0.452	55.2 ± 9.6	55.8 ± 8.9	0.875
Glu [mg/dl]	131.2 ± 78.4	157.1 ± 101.4	100.8 ± 18.9	0.210	277.0 ± 111.7	104.6 ± 33.1	0.269
Protein [g/dl]	7.3 ± 0.6	7.4 ± 0.6	7.2 ± 0.6	0.636	6.6 ± 0.0	7.3 ± 0.6	0.230
Bilirubin [mg/dl]	1.1 ± 1.4	0.9 ± 0.9	1.3 ± 1.7	0.611	1.7 ± 1.4	1.0 ± 1.3	0.336
AST [U/l]	73.4 ± 116.8	46.3 ± 43.6	96.6 ± 152.9	0.258	59.8 ± 34.4	75.9 ± 126.6	0.805
ALT [U/l]	80.8 ± 179.9	52.5 ± 77.4	109.2 ± 244.3	0.433	62.5 ± 48.8	84.1 ± 195.2	0.830
ALP [U/l]	121.5 ± 100.5	122.5 ± 115.8	120.2 ± 84.6	0.959	207.5 ± 184.4	102.3 ± 65.9	0.338
GGT [U/l]	225.1 ± 614.2	113.3 ± 170.3	322.1 ± 825.2	0.380	263.0 ± 260.9	218.8 ± 658.5	0.897
CRP [mg/l]	23.2 ± 42.3	39.8 ± 60.4	11.4 ± 16.6	0.178	63.4 ± 104.2	17.4 ± 26.3	0.525
Helicobacter (%)	21.9	23.1	21.1	0.892	0	25.0	0.258

*ALP* alkaline phosphatase, *ALT* alanine aminotransferase, *AST* aspartate aminotransferase, *Cr* creatinine, *CRP* C-reactive protein, *eGFR* estimated glomerular filtration rate, *GGT* gamma-glutamyl transferase, *Glu* glucose, *Hb* haemoglobin, *Hct* haematocrit, *INR* International Normalized Ratio, *MCH* mean corpuscular haemoglobin, *MCHC* mean corpuscular haemoglobin concentration, *MCV* mean corpuscular volume

### Histological examination of biopsies from the upper gastrointestinal tract

**Fig. 1:**
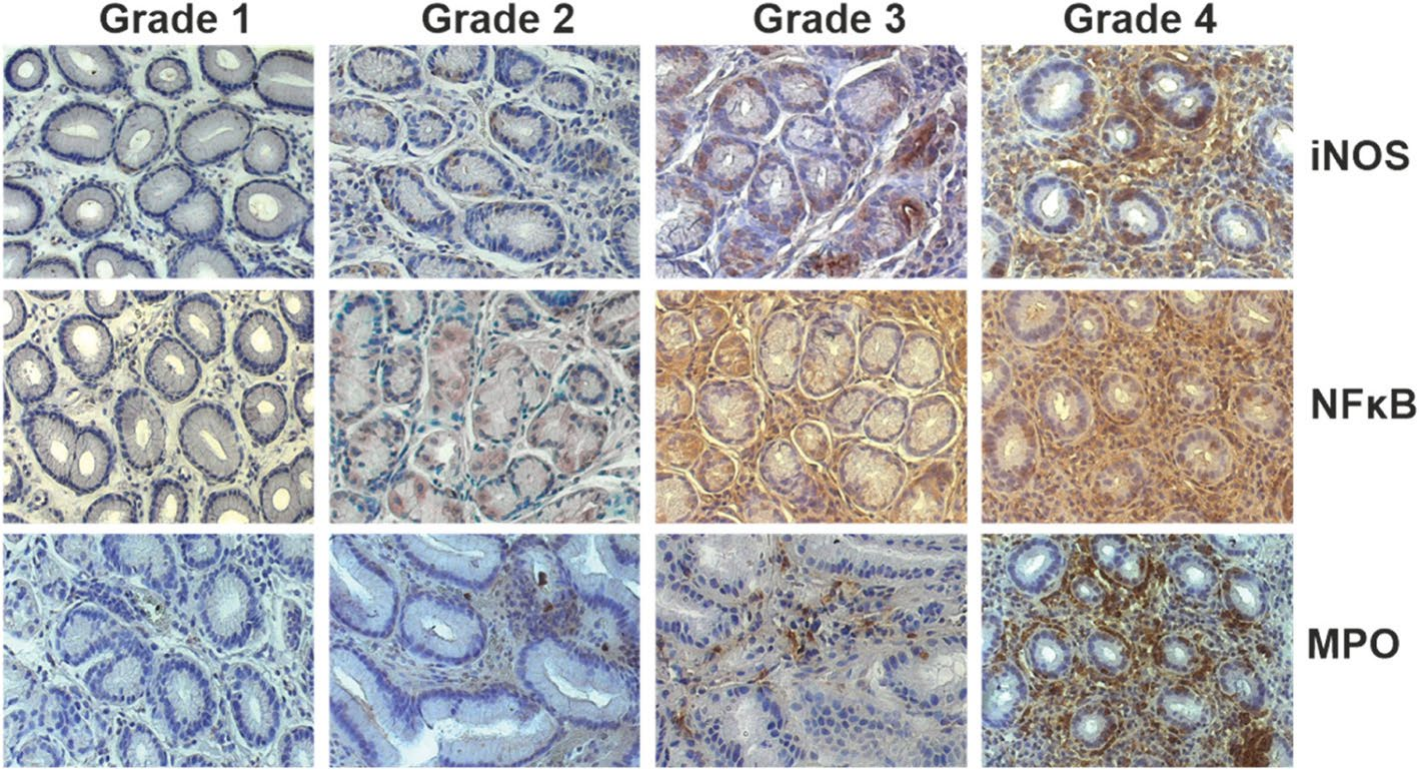
Histological classification of biopsies from the mucosa of the upper gastrointestinal tract showing the expression of iNOS, NF-κ B and MPO, as assessed by means of immunohistochemical staining using specific antibodies

Immunohistochemical sections from the biopsies showed broad staining patterns since all four different scores were observed in the sample which ranged from the absence of any immunopositivity in biopsies with no histological features of inflammation (grade 1) to the highest expression level as assessed by an intense positive immunoreactivity (grade 4). Figure 1 and Table 2 show the data from the immunohistochemical scoring of the three inflammation markers iNOS, NF-κ B and MPO in the gastric biopsies. In the study sample, all markers were graded from 1 to 4, with high numbers of samples scoring in grades 1 and 2, respectively. In contrast, the number of patients classified as grade 4 was low for all three markers, indicating that the majority of participants had only slight to moderate inflammation or even no detectable signs of infiltration.

**Table 2:** Histological expression grade of inflammatory proteins in gastric biopsies from a sample of patients with upper gastrointestinal symptoms

	Grade 1	Grade 2	Grade 3	Grade 4
iNOS	6	14	7	5
NF-κ B	15	10	5	2
MPO	12	11	5	4

*MPO* Myeloperoxidase, *NF*-κ* B* Nuclear factor kappa-light-chain-enhancer of activated B-cells, *iNOS* Inducible nitric oxide synthase

**Fig. 2:**
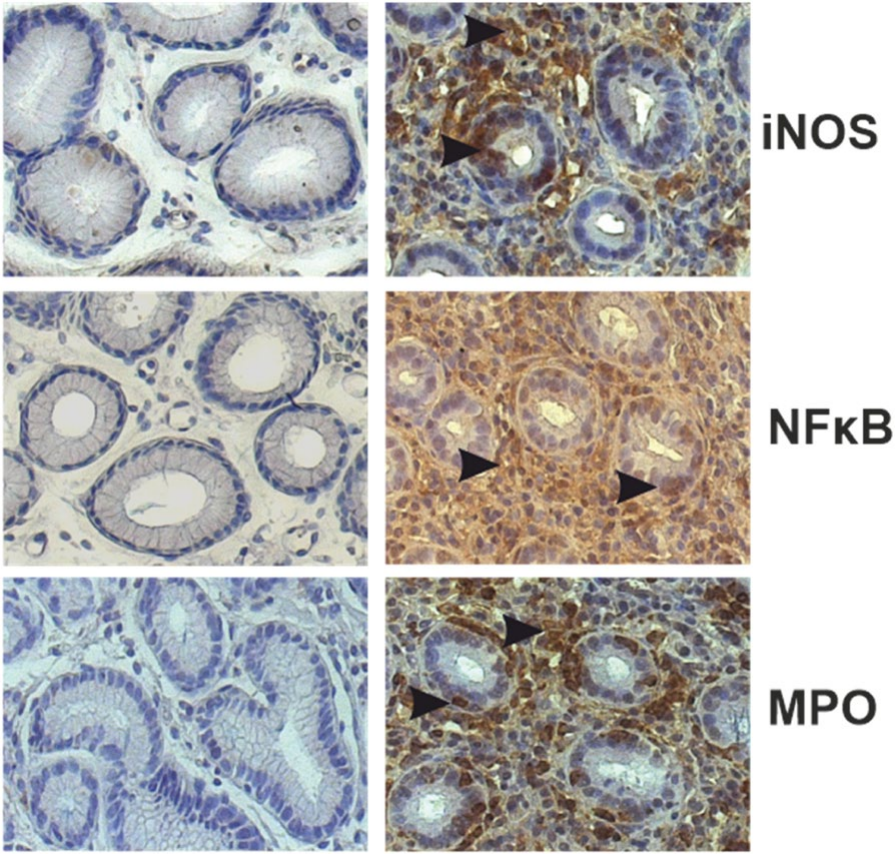
Immunohistological staining of iNOS, NF-κ B and MPO in biopsies of the upper gastrointestinal tract (right panel) of patients undergoing gastroscopy. Arrow heads mark areas of positive immunoreactivity. Left panel: Negative controls in the absence of inflammatory infiltrates

**Fig. 3:**
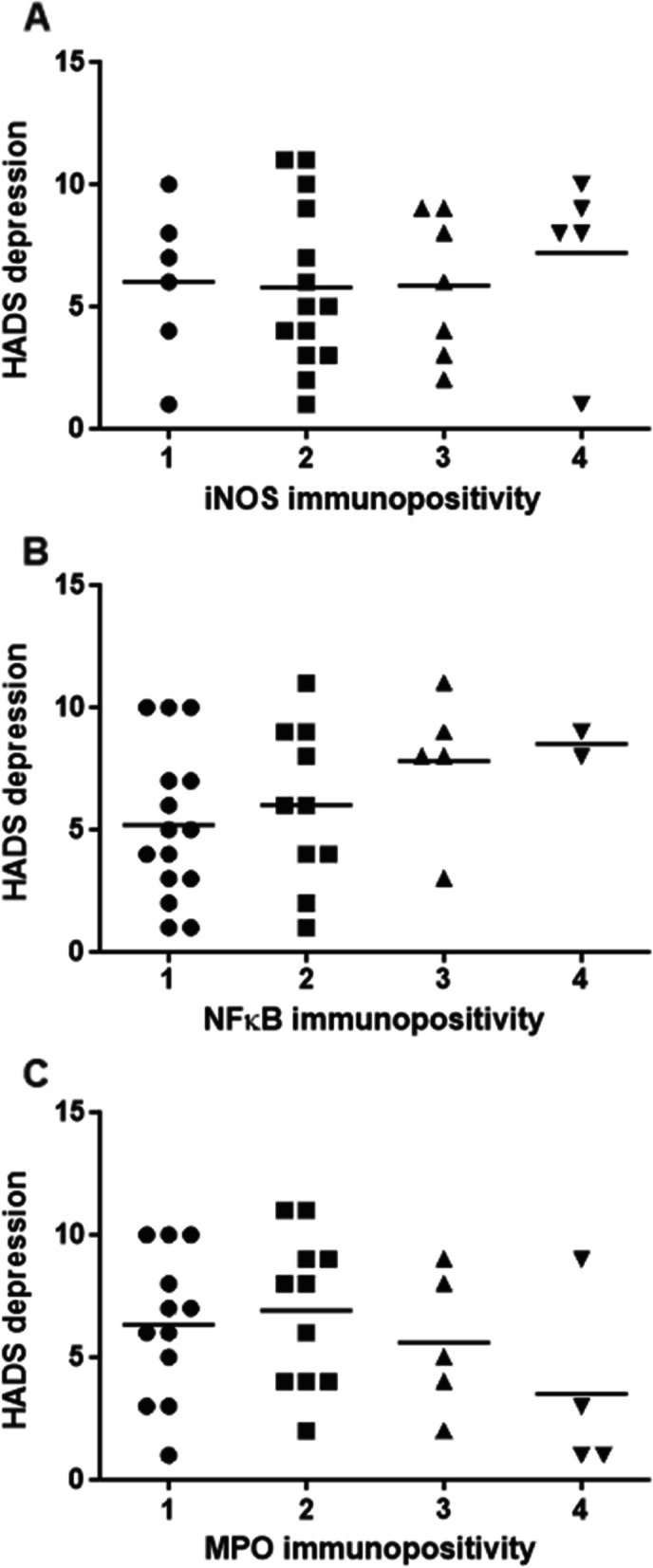
Distribution of HADS-D scores by classes of immunopositivity for iNOS (A), NF-κ B (B) and MPO (C). Whereas iNOS and MPO expression were unrelated to HADS depression, there was a steady and statistically significant increase in HADS depression scores along the four groups of increasing NF-κ B expression (*p* = 0.032)

Expression of iNOS was most prominent in infiltrating immune as well as stromal cells, but was also detectable, although to a lesser extent, in the lining epithelial cells of the *foveolae gastricae*. In sections with mild inflammation, there was a predominant staining of epithelial cells. Immunolabelling with NF-κ B antibodies revealed a similar staining pattern, with a particularly high immunoreactivity in inflammatory infiltrates. Expression of NF-κ B was also located in cells of the subepithelial connective tissue (Fig. 2). In contrast, staining with anti-MPO antibodies was mainly restricted to infiltrating immune cells such as macrophages, whereas epithelial cells did not exhibit a significant and specific staining pattern (Figs. 2 and 3). Among the samples, there was a significant and positive correlation between the expression grade of NF-κ B and iNOS (*r* = 0.61, p < 0.001), while the expression of MPO neither correlated with NF-κ B (*n* = 0.340) nor iNOS (*p* = 0.318).

### Association of histochemical results and HADS depression

In addition, we assessed the relationships between HADS depression or anxiety with the immunohistopathological expression levels of the three inflammatory biomarkers present in gastritis. To this end, we first tested whether there were significant differences in the expression levels of each of the markers between study participants with normal and elevated scores for depression and anxiety above the clinically relevant cut-off threshold (Fig. 3, Table 3). In the group comparison of the study participants with normal versus elevated HADS-D scores, there was a statistically significant difference in the mean NF-κ B expression levels. Patients with an elevated HADS score displayed a more prominent NF-κ B immunoreactivity compared to their counterparts with no self-rated depressive symptoms (2.4 ± 1.0 vs. 1.4 ± 0.6, *p* = 0.008). In contrast, anxious and non-anxious patients did not differ with respect to their NF-κ B expression levels (*p* = 0.674). Furthermore, our data showed that gastric iNOS and MPO expression levels were within the same range for depressed and non-depressed patients, as was also observed for HADS anxiety scores (Table 3). Group comparisons between the four classes of NF-κ B immunoreactivity using ANOVA demonstrated significant differences between the MCV of red blood cells (*p* = 0.008) and the mean corpuscular haemoglobin content (MCH, *p* = 0.004), as shown in Table 4. The detection of *H. pylori* was unrelated to the mucosal expression of NF-κ B (*p* = 0.804), iNOS (*p* = 0.124), and MPO (*p* = 0.256).

**Table 3:** Expression level of iNOS, NF-κ B, and MPO in biopsies from the upper gastrointestinal tract from patients with normal and elevated HADS scores for depression and anxiety. Shown are the means and standard deviations of the expression grade for iNOS, NF-κ B and MPO in patients with normal and elevated HADS-D or HADS-A scores as well as the corresponding *p* values for the comparisons between the two groups

	Total study population (*n* = 32)	Subjects with elevated HADS-D score (*n* = 13)	Subjects with normal HADS-D score (*n* = 19)	*P*-value	Subjects with elevated HADS-A score (*n* = 4)	Subjects with normal HADS-A score (*n* = 28)	*P*-value
iNOS	2.3 ± 1.0	2.7 ± 1.1	2.1 ± 0.8	0.093	2.5 ± 1.3	2.3 ± 0.9	0.737
NF-κ B	1.8 ± 0.9	2.4 ± 1.0	1.4 ± 0.6	**0.008**	2.0 ± 0.8	1.8 ± 1.0	0.674
MPO	2.0 ± 1.0	2.0 ± 0.9	2.1 ± 1.1	0.890	1.5 ± 0.6	2.1 ± 1.1	0.278

*MPO* Myeloperoxidase, *iNOS* Inducible nitric oxide synthase, *NF*-κ* B* Nuclear factor kappa-light-chain-enhancer of activated B-cells

**Table 4:** Associations of NF-κ B expression levels with clinical parameters. Shown are frequencies (%) or means and standard deviations of the indicated variables as well as their corresponding *p* values for the comparisons between the four groups of mucosal NF-κ B expression levels

	NF-κ B immunopositivity				
	1	2	3	4	*P*-value
Sex [male, %]	67	50	100	50	0.292
Age [years]	56.1 ± 11.8	51.1 ± 17.1	55.0 ± 14.9	54.5 ± 29.0	0.801
Quick	103.1 ± 20.4	87.6 ± 19.1	86.8 ± 17.5	97 ± 0.0	0.262
INR	1.0 ± 0.1	1.1 ± 0.2	1.1 ± 0.2	1.0 ± 0.0	0.432
MCV [fl]	91.9 ± 5.0	82.4 ± 8.0	90.0 ± 4.4	87.5 ± 2.1	**0.008**
MCH [pg]	30.8 ± 1.8	27.1 ± 2.9	30.2 ± 1.8	28.9 ± 1.7	**0.004**
MCHC [g/dl]	33.5 ± 0.7	32.9 ± 0.9	33.6 ± 0.8	33.1 ± 1.0	0.281
Platelets [10^3^/µl]	218.7 ± 79.7	227.2 ± 123.7	242.6 ± 49.2	533.0 ± 76.4	**0.001**
Leukocytes [10^3^/µl]	6.9 ± 2.7	6.4 ± 2.1	8.1 ± 1.3	9.4 ± 3.4	0.322
Na^+^ [mmol/l]	138.7 ± 1.8	136.5 ± 5.3	141.4 ± 2.3	137.5 ± 6.4	0.114
K^+^ [mmol/l]	3.9 ± 0.4	4.2 ± 0.4	3.7 ± 0.2	4.9 ± 1.3	**0.016**
Creatinine [mg/dl]	0.9 ± 0.4	1.1 ± 0.5	0.9 ± 0.4	0.6 ± 0.2	0.408
MPO	1.9 ± 1.2	2.1 ± 0.9	2.2 ± 1.1	2.5 ± 0.7	0.823
iNOS	1.9 ± 0.7	2.4 ± 1.0	3.0 ± 0.7	4.0 ± 0.0	**0.004**

*MPO* Myeloperoxidase, *iNOS* Inducible nitric oxide synthase, *NF*-κ* B* Nuclear factor 'kappa-light-chain-enhancer' of activated B-cells

Finally, a logistic regression model was computed with abnormal HADS depression score above the cut-off level as the dependent variable adjusted to sex, age and MCV to confirm the association of the histopathologically measured NF-κ B expression level with self-rated depression symptoms. Data showed that the expression of the transcription factor NF-κ B was significantly and positively linked to abnormal HADS depression (Exp(β) = 4.51, 95% confidence interval 1.32–15.40, *p* = 0.016, Table 5).

**Table 5:** Results from a binary logistic regression model with elevated depression scores (HADS-D ≥ 8) as dependent variable and NF-κ B as independent variable adjusted for the following confounders: sex, age, and MCV (R^2^ = 0.325; *p* = 0.016)

	Exp(B)	95%-CI	Wald	*p*-value
Sex	1.206	0.154 – 9.426	0.032	0.858
Age	1.053	0.979 – 1.132	1.924	0.165
MCV	0.939	0.819 – 1.076	0.824	0.364
NF-κ B	4.514	1.319 – 15.450	5764	**0.016**

*MCV* Mean corpuscular volume, *NF*-κ* B* Nuclear factor 'kappa-light-chain-enhancer' of activated B-cells

## Discussion

The present study confirms the high prevalence of comorbid depression, as assessed by positive HADS depression scores above a clinically relevant cut-off, in patients who had undergone an elective gastroscopy for upper gastrointestinal complains. The main finding of this study is the positive association between self-rated depressive symptoms and the expression level of the transcription factor NF-κ B in biopsies from the upper gastrointestinal tract. The link to the degree of mucosal NF-κ B expression was restricted to HADS depressive symptoms, as there was no indication of an association between psychometrically detected anxiety scores and NF-κ B expression in the immunohistochemical samples from the gastric mucosa.

Previous clinical studies have documented elevated levels of depressive symptoms among selected samples of patients with gastritis [4, 5] and high rates of mood and anxiety disorder diagnoses among general gastroenterology outpatients [2, 3]. The prevalences of depression and anxiety are higher in patients with chronic diseases compared to the general population, and having a long-term medical illness is a risk factor for depression [18].

Despite extensive efforts in the last few years, the neurobiological mechanisms underlying depression have not yet been fully characterized. It is well documented that genetic factors contribute to depression and, in addition, environmental factors such as traumatic or repeated stress exposure can trigger or exacerbate mental mood disorders [19–21]. In addition, several studies have shown that depression is associated with increased proinflammatory cytokine levels [22, 23] and that the transcription factor NF-κ B plays an important role in inflammatory processes [24, 25]. NF-κ B is highly activated at sites of mucosal inflammation where it contributes to the development of gastritis and induces transcription of proinflammatory cytokines, such as tumor necrosis factor-α (TNF-α), IL-1β, IL-6, and IL-8, as well as chemokines, adhesion molecules, matrix metalloproteinases (MMPs), Cox-2, and iNOS [8, 26].

*H. pylori*-associated gastritis is marked by increased NF-κ B activity in gastric epithelial cells and the number of NF-κ B positive cells correlates with the degree of gastritis [7]. The activation of NF-κ B may in turn aggravate gastritis via the induction of proinflammatory cytokines such IL-1, IL-6, IL-8 and TNF-α [8, 27]. In line with these findings, our results showed a detectable NF-κ B expression in biopsies from the upper gastrointestinal tract and, moreover, the intensity of this NF-κ B immunoreactivity was significantly and positively associated with elevated HADS depression scores. However, in our small sample, we did not observe an increased NF-κ B expression in *H. pylori*-associated gastritis as compared to other, non-infectious causes of gastric inflammation. The incidence of *H. pylori*-associated gastritis in our study population (21.9%) was within the range reported from other European surveys [28].

Little is known about the mechanisms converting psychosocial stress into cellular dysfunction, but various genes up-regulated by psychosocial stress are controlled by the transcription factor NF-κ B [29]. Many observations have shown that effects of the cytokine system on serotonin metabolism as well as on the hypothalamic–pituitary–adrenal (HPA) axis may induce changes in the structure and function of the brain, possibly leading towards the development of depression [24, 29]. In line with our results, Bierhaus and colleagues showed that activation of NF-κ B represents a downstream effector of the neuroendocrine response to stressful psychosocial events, such as depression, and triggers changes in neuroendocrine axis activity in cellular responses [30].

Psychological stress plays a pivotal role in the etiology of both gastric inflammation and depressive syndrome. In humans, chronic stress alters various physiological pathways resulting in elevated glucocorticoid levels, increased oxidative stress, reduced neurotransmitter levels, and inflammatory reactions in the gastric mucosa [31]. In adult rats it was shown that exposure to chronic unpredictable stress impaired hippocampal neurogenesis and promoted anxious, depression-like behaviors. Chronic unpredictable stress led to increased gliosis and activation of microglia as well as the induction of neuronal apoptosis in both the cerebral cortex and the hippocampal region in stress-exposed rodents [32, 33]. Our clinical data suggest a link between the development of stress-induced gastric mucosal lesions and the induction of depressive symptoms, which is an indicator of an underlying mild inflammatory reaction both in the brain and the stomach.

A limitation of our study is related to the fact that depression was measured only by psychometric self-rating and not by structured clinical interviews. Thus, we cannot conclude whether our results can be generalized to specific depressive disorders or mainly reflect non-specifically increased depression preoccupation. However, the HADS instrument used in this investigation has previously been demonstrated to be particularly valuable for the detection of depression and anxiety in physically ill patients. Furthermore, the data are based on a cross-sectional analysis, as repeated biopsies and reassessment of depressive symptoms were not performed. Thus, time-dependent covariates or repeated measures could not be included in the analysis due to the lack of a longitudinal study design. Similarly, it was not possible to establish a cause-and-effect relationship. The small effect size limits the clinical and diagnostic implications of these novel findings. One reason for this is the possible influence of the gastrointestinal microbiome on gastrointestinal immunity. Since cohort effects cannot be empirically excluded, the results from the study must not be interpreted as statistically certain. Additionally, the cohort size between depressed (*n* = 13) and non-depressed patients (*n* = 19) was unbalanced and very small.

However, the study has also distinct strengths which include the heterogeneous study population with well-defined, complete clinical and histopathological data. In addition, commercially available antibodies with high diagnostic accuracy were used for immunohistochemical staining and the psychometric screening instruments used were well-validated. The results from our pilot study warrant replication in an independent sample of patients with upper gastrointestinal symptoms including long-term follow-up. Certainly, further experiments will clarify the role of NF-κ B in gastrointestinal inflammatory tissues in relation to comorbid depressive symptoms.

In summary, in this prospective monocentric cohort study we demonstrate that the expression of NF-κ B in biopsies of the upper gastrointestinal tract is associated with clinically relevant depression as assessed by a self-rated questionnaire. However, considerably more research is required to confirm these findings and decipher possible physiological mechanisms which may underlie this association.

## Data Availability

Data underlying this article will be shared on reasonable request to the corresponding author.

## Abbreviations

ANOVA: Analysis of variance
COX-2: Cyclo-oxygenase-2
HADS: Hospital Anxiety and Depression Scale
iNOS: Inducible nitric oxide synthase
MCH: Mean corpuscular haemoglobin
MCV: Mean corpuscular volume
MPO: Myeloperoxidase
NF-κ B: Nuclear factor kappa B
